# Supplementary material for: Ambient carbon dioxide concentration correlates with SARS-CoV-2 aerostability and infection risk
Source: Nat Commun. 2024 Apr 25;15:3487. doi: 10.1038/s41467-024-47777-5 (PMC11045827; doi:10.1038/s41467-024-47777-5)
Supplement: Supplementary file 1 — Supplementary Information [file 41467_2024_47777_MOESM1_ESM.pdf]

Supplemental Information

Supplementary Results

Supplemental Table 1: Number of Replicates for Each Measurement in Figures 1 to 3

Figure	Sample	X-Axis	Y-Axis	SD	n	SE
1A	Delta (RH= 90, 500 ppm)	5	1	0.34127	24	0.06966
		10	1.03725	0.56392	4	0.28196
		15	1.01834	0.37444	8	0.13238
		30	0.67269	0.37488	8	0.13254
		60	0.48762	0.18485	8	0.06535
		120	0.36961	0.17336	12	0.05005
		300	0.27476	0.18499	11	0.05578
1A	Delta (RH=40, 500 ppm)	0	1			0
		5	0.4534	0.12618	7	0.04769
		30	0.5195	0.22135	8	0.07826
		120	0.29237	0.16849	5	0.07535
		300	0.34334	0.20063	9	0.06688
1A	Omicron (RH=90, 500ppm)	5	1	0.30404	9	0.10135
		30	0.57877	0.23988	9	0.07996
		120	0.52251	0.15244	17	0.03697
		300	0.39003	0.20684	12	0.05971
1A	Omicron (RH=40, 500ppm)	30	0.45448	0.26207	7	0.09905
		120	0.50838	0.34227	6	0.13973
		300	0.51583	0.20911	7	0.07904
1B	Omicron	0	100	0	3	0
	Omicron	600	79.32317	12.83216	3	7.40865
	Omicron	1200	57.66003	5.97658	3	3.45058
	Omicron	1800	57.60721	17.11193	3	9.87958
1B	Delta	0	100	9.62E-09	3	5.56E-09
	Delta	600	23.28	2.63445	3	1.521
	Delta	1200	8.592	2.19797	3	1.269
	Delta	1800	2.167	1.53633	3	0.887
1C	Omicron	0	100	0	3	0
	Omicron	600	99.65	1.47224	3	0.85
	Omicron	1200	87.85	9.43968	3	5.45
	Omicron	1800	93.45	10.1325	3	5.85
	Omicron	3600	92.4	6.9282	3	4
1C	Delta	0	100	0	3	0
	Delta	600	51.24333	17.02738	3	9.83076
	Delta	1200	34.99	8.74111	3	5.04668
	Delta	1800	24.73333	5.64127	3	3.25699
	Delta	3600	22.31667	6.98645	3	4.03363
2A	Delta	0	42.09488	26.73879	24	0.05458
	Delta	550	0.36961	0.17336	12	0.05005
	Delta	800	66.604	19.66615	14	5.256
	Delta	1800	75.031	29.94197	7	11.317
	Delta	3050	73.759	35.87294	8	12.683
	Delta	6372	77.626	29.825	25	5.965
	Beta	500	57.989	22.79571	18	5.373
	Beta	3000	81.392	2.12523	3	1.227
	Omicron	500	54.55	15.635	25	3.127
	Omicron	3000	66.275	23.60008	10	7.463
2B	500 ppm	15	101.834	37.44381	8	13.23839
	500 ppm	30	67.26854	37.48761	8	13.25387
	500 ppm	120	36.96071	17.33642	12	5.00459
	500 ppm	300	27.47563	18.49853	11	5.57752
	500 ppm	1200	8.51536	6.77181	3	3.9097
	500 ppm	2400	3.19792	3.49205	3	2.01614
	3000 ppm	15	92.0054	41.93496	51	5.87207
	3000 ppm	30	78.68192	30.07776	5	13.45118
	3000 ppm	120	73.75874	35.87317	8	12.68308
	3000 ppm	300	53.42633	21.7061	4	10.85305
	3000 ppm	600	44.16785	29.05119	5	12.99209
	3000 ppm	1200	30.92149	18.496	7	6.99083
	3000 ppm	2400	26.12552	15.37162	6	6.27544
	6000 ppm	15	87.07512	37.26751	18	8.78404
	6000 ppm	30	87.5809	45.61365	7	17.24034
	6000 ppm	120	77.62563	29.82572	25	5.96514
	6000 ppm	300	54.32607	25.22585	7	9.53448

Figure	Sample	X-Axis	Y-Axis	SD	n	SE
2C		15	0.90348		8	0.13084
		30	1.16967		8	0.30512
		120	1.9956		12	0.43677
		300	1.9445		11	0.55843
		1200	3.63126		3	1.85841
		2400	8.16953		3	5.51166
2D	Bulk	0	4.60517		3	2.56E-10
	Bulk	10	3.14759		3	0.04787
	Bulk	20	2.15083		3	0.02729
	Bulk	30	0.77334		3	0.00686
	500 ppm	0				
	500 ppm	0.25	4.62334		8	0.61206
	500 ppm	0.5	4.20869		8	0.55781
	500 ppm	2	3.60986		12	0.18066
	500 ppm	5	3.3133		11	0.1848
	500 ppm	20	2.14187		3	0.08374
	500 ppm	40	1.1625		3	0.02344
	3000 ppm	15	4.52185		51	0.26553
	3000 ppm	30	4.36541		5	0.5872
	3000 ppm	120	4.3008		8	0.54547
	3000 ppm	300	3.9783		4	0.43177
	3000 ppm	600	3.788		5	0.49214
	3000 ppm	1200	3.43145		7	0.23989
	3000 ppm	2400	3.26291		6	0.20476
3A	Omicron	20	54.37	18.4206	9	6.14
	Omicron	30	44.967	21.5932	11	6.511
	Omicron	40	70.248	18.915	10	5.981
	Omicron	50	66.328	25.1195	10	7.943
	Omicron	60	98.108	16.2989	9	5.433
	Omicron	80	94.259	25.655	5	11.473
	Omicron	90	100			13.238
	Delta	20	37.62866	17.90545	8	6.33053
	Delta	40	45.17823	5.21531	4	2.60766
	Delta	60	34.7452	21.15787	9	7.05262
	Delta	80	53.98153	18.87624	6	7.70619
	Delta	90	92.0054	41.93496	51	5.87207
3B	500 ppm	20	37.62866	17.90545	8	6.33053
	500 ppm	40	45.17823	5.21531	4	2.60766
	500 ppm	60	34.7452	21.15787	9	7.05262
	500 ppm	80	53.98153	18.87624	6	7.70619
	500 ppm	90	92.0054	41.93496	51	5.87207
	3000 ppm	20	71.36276	29.47005	8	10.41924
	3000 ppm	40	71.81949	24.10744	6	9.84182
	3000 ppm	60	66.30721	19.39388	5	8.67321
	3000 ppm	80	103.5697	29.29396	6	11.95921
	3000 ppm	90	95.62162	37.0697	26	7.26997
	6000 ppm	20	63.72523	14.3458	6	5.85665
	6000 ppm	40	73.37041	3.84388	4	1.92194
	6000 ppm	60	54.75939	15.06845	6	6.15167
	6000 ppm	80	100.0452	26.29354	6	10.73429
	6000 ppm	90	87.07512	37.26751	18	8.78404

The solute composition of saliva between individuals may vary widely. Moreover, an individual's salivary [NaCl], as well as the ratio of NaCl to other solutes, may vary dramatically as a result of short-term events, such as oral stimulation [1], or larger physiological changes, such as pregnancy [2]. The effect that altering the initial [NaCl] of the starting formulation on the short term aerostability is reported in Supplemental Figure 1.

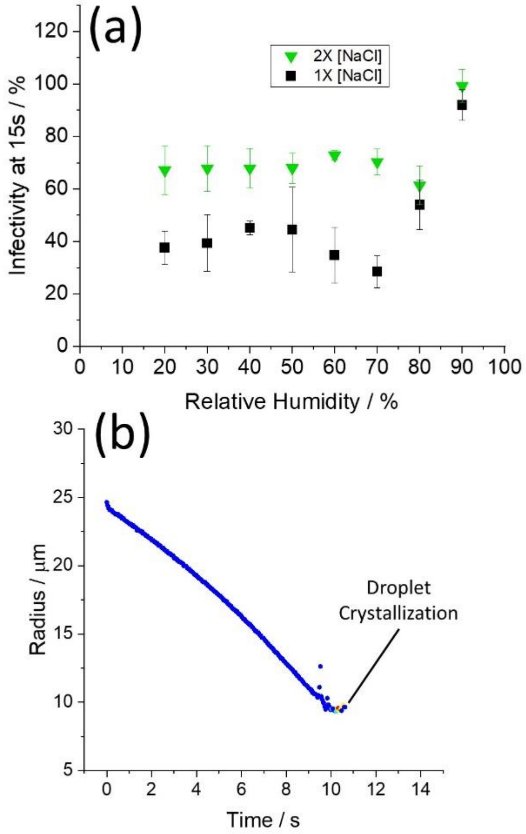

Supplemental Figure 1: Effect of NaCl concentration on (A) aerostability of SARS-CoV-2 across a range of RH and (B) droplet evaporation dynamics. (A) Infectivity of the Delta variant at 15 seconds as a function of relative humidity and the [NaCl] in the droplet. Error bars indicate the standard error. A two-way ANOVA of the data in indicated that [NaCl] had a significant effect ($P < 0.0001$), with no significant interaction between the RH and [NaCl]. (B) The evaporation profile of MEM droplets containing 2X the amount of NaCl when injected into an airflow where the RH was 69% measured with a comparative kinetic electrodynamic balance (CK-EDB) as described previously [3]. Once the droplet becomes a non-spherical/non-homogeneous/amorphous solid, the droplet is no longer spherical and thus cannot be sized using Mie theory.

We previously reported that the fraction of SARS-CoV-2 in a particle that resides in the salt crystal following the efflorescence event is shielded and thus remains infectious. Accordingly, any observed increase in sustained infectivity below an RH of 50% can be attributed to the increase in the volume of the particle that forms a salt crystal. The same trend is observed in Supplemental Figure 1A: the addition of NaCl results in a significant increase in sustained aerostability at RHs below 50%. Between the RHs

of 50% and 75%, we previously reported that the physical structures of MEM droplets is complex with phase structures across a population of droplets that are a mixture of a homogeneous liquid droplet form and liquid droplets containing a suspension (e.g. emulsion or salt crystals) [4]. When the [NaCl] is doubled, all particles undergo a significant phase change when the RH is below ~75% (Supplemental Figure 1B). Accordingly, the crystalline form offers protection to the virus when the RH is below ~75% and this protection is more pronounced when the [NaCl] is doubled. Hence, the remaining fraction of infectious virus remains higher across all RHs below 75%. At 80% RH, no phase change is observed and the increase of [NaCl] has no effect on the viral aerostability.

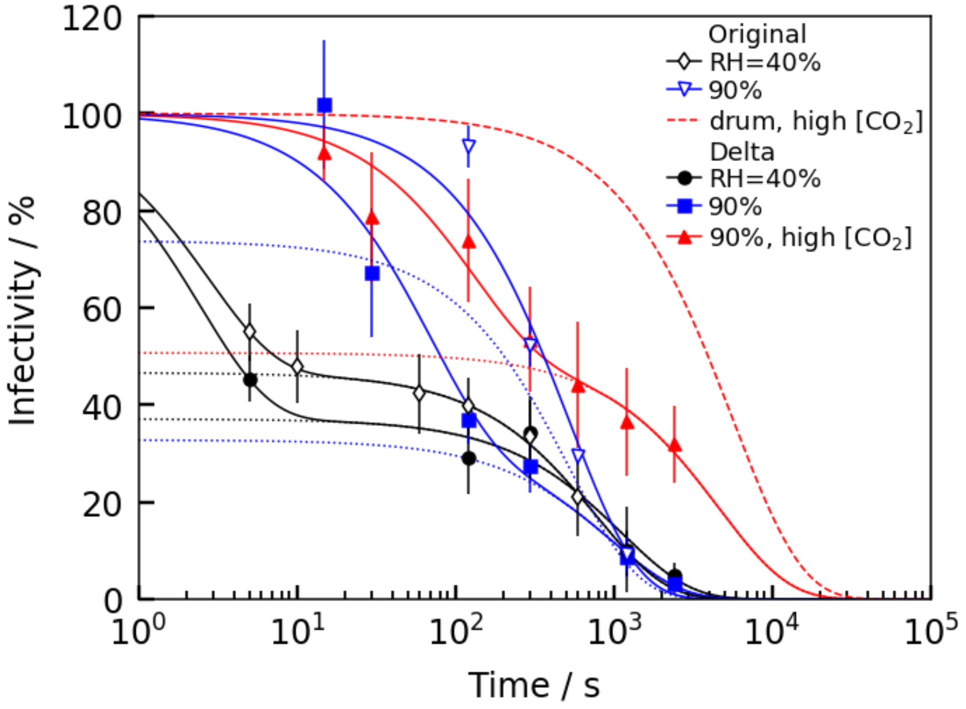

Supplemental Figure 2: Decay profile fits to the CELEBS infectivity data with two exponentially decaying functions, to capture the distinct short and long decays. We show the complete fits of the data from a two-step exponential decay (solid lines), and also just the longer time part of this fit (dotted lines). We show also the standard 1.1hr decay assumed in the literature from drum studies (dashed red).

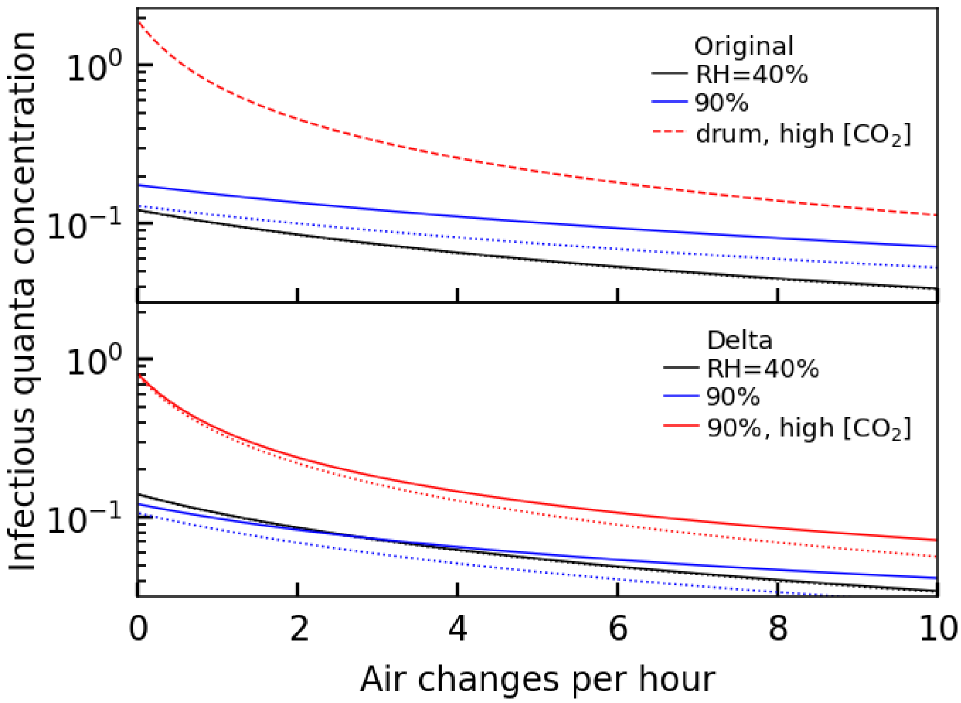

Supplemental Figure 3: Concentration of infectious quanta in a 300m^3 classroom using two-step decay fits to CELEBS data (solid lines) and just the longer time one-step decay (dotted lines). The incomplete latter fit captures the behavior very well at 40% RH, but deviates at 90% because the two-timescales in the complete fit are not neatly separated. We show also the standard 1.1hr decay assumed in the literature from drum studies (dashed red in top panel).

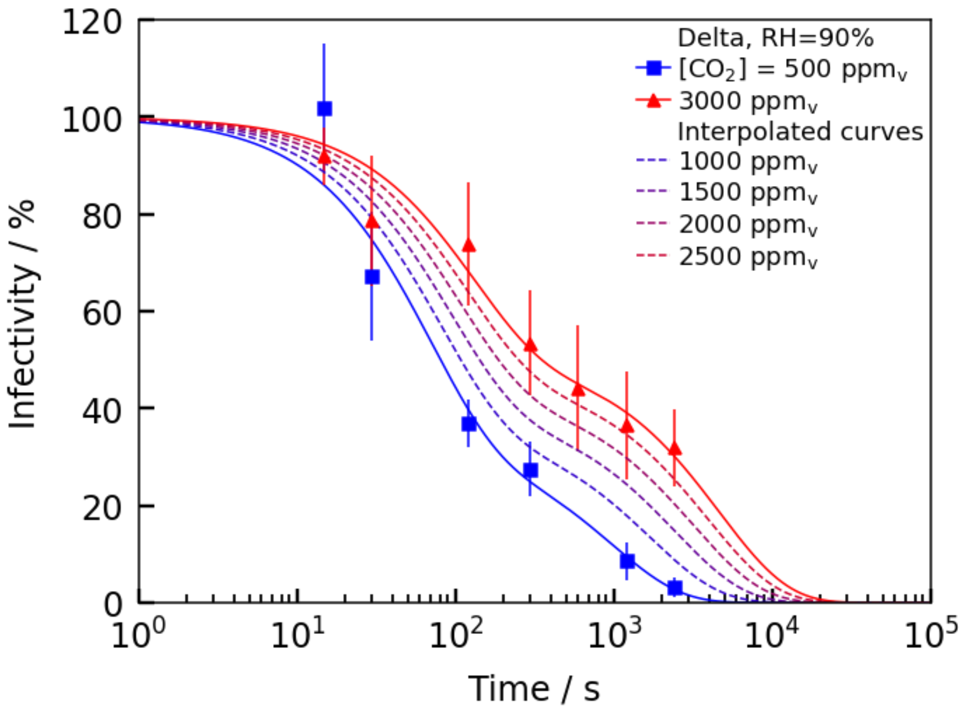

Supplemental Figure 4: Delta data fits (solid lines) and their crude linear interpolation across CO_2 concentration for illustration purposes (dotted lines).

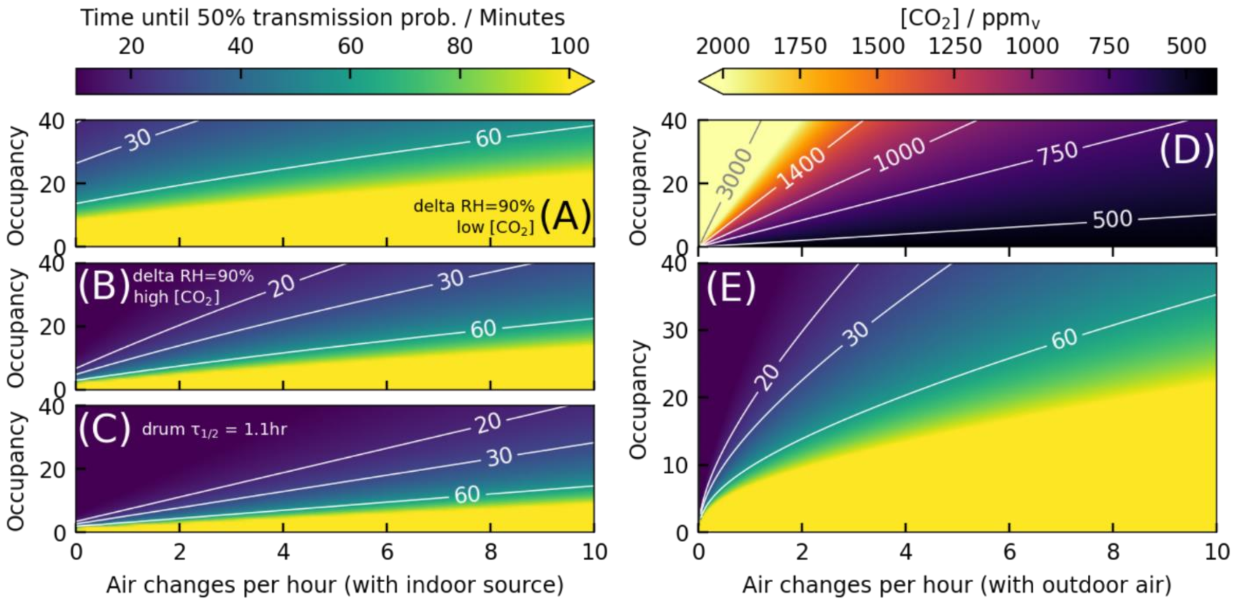

Supplemental Figure 5: (A-C) Time until there is a 50% chance that at least one susceptible person will have become infected in an occupied classroom containing a single infected individual. We neglect the coupling between ventilation and [CO_2], instead using the fits to the data at fixed [CO_2], so this models air exchange with an interior source. (D) Variation in [CO_2] with occupancy and ventilation. (E) Sketch of how factoring in CO2 changes the time using the linear interpolation shown in Supplemental Figure 4 for delta at 90% RH. This interpolation is purely intended to illustrate the nonlinear role of ventilation qualitatively, and so the numbers in (E) should not be taken literally. An infectious quanta production rate of $0.1s^{-1}$ is assumed in (A-C,E).

Fits of experimental data

For the Wells-Riley calculation, we fit each experimental dataset to a double exponential decay model which is more convenient for calculations. Specifically, we perform a least-squares fit of the infectivity to

$$I(t) = Aexp\left(\frac{-t}{\tau_1}\right) + (1-A)exp\left(\frac{-t}{\tau_2}\right)$$

where t is time, τ_1 and τ_2 are characteristic decay times, and A is a dimensionless factor representing the relative amplitude of each decay mode. The first term represents an initial short-time decay, whereas the second term represents slower decay at longer times. In Supplemental Figure 2 we show the results of fitting the raw data (solid lines) and just the slower decay at longer times (dotted lines) to show the relative size of the second mode.

Infectious quanta concentration

For the Wells-Riley calculations we must calculate the expected concentration of infectious quanta in the well-mixed environment. A droplet produced at some time Δt before the present will still be around and infectious with probability $p_{active}\, I$. The total number of droplets still around at time t is then obtained by integrating over all $\Delta t>0$ i.e. over all the past. Writing α as the rate of production, we therefore find the concentration is:

$$c = \alpha \int_0^\infty p_{active}(\Delta t) I(\Delta t) d\Delta t$$

We show the result of this calculation in Supplemental Figure 3 for our assumed quanta production rate of $\alpha = 0.1s^{-1}$.

Varying CO_2

For the Delta variant at 90% relative humidity we have two datasets at CO_2 concentrations of 500ppm and 3000ppm. To obtain use data at intermediate concentrations for illustration purposes we interpolate the fit parameters (A, τ_1, τ_2) and insert them into the same fitting function. The results of this interpolation are shown in Supplemental Figure 4.

To estimate the CO_2 concentration in a room of N occupants at fixed volume, we again assume the air is well-mixed so the concentration is uniform and that the CO_2 produced by the occupants is constant. We take the average per-person CO_2 production rate typical of a high school classroom as R=0.0065l/s [2]. The expected $[CO_2]$ is then

$$[CO_2] = [CO_2]_0 + N\frac{R}{Q}$$

where Q is the volumetric flow rate out of the room, and we take the background $[CO_2]_0$ as 420ppm for direct exchange with outdoor air. The resulting concentration for the 300m3 classroom with different occupancies and ventilation rates is shown in Supplemental Figure 5D.

Finally, we calculate the time until the risk of transmission reaches 50% for occupancies in the range of 0-40 at fixed $[CO_2]$ in Supplemental Figure 5A-C, and with varying $[CO_2]$ using the interpolations of Supplemental Figure 4 and the calculation above for the $[CO_2]$. These contour plots show the same data as in the main text but across the full range of occupancies.

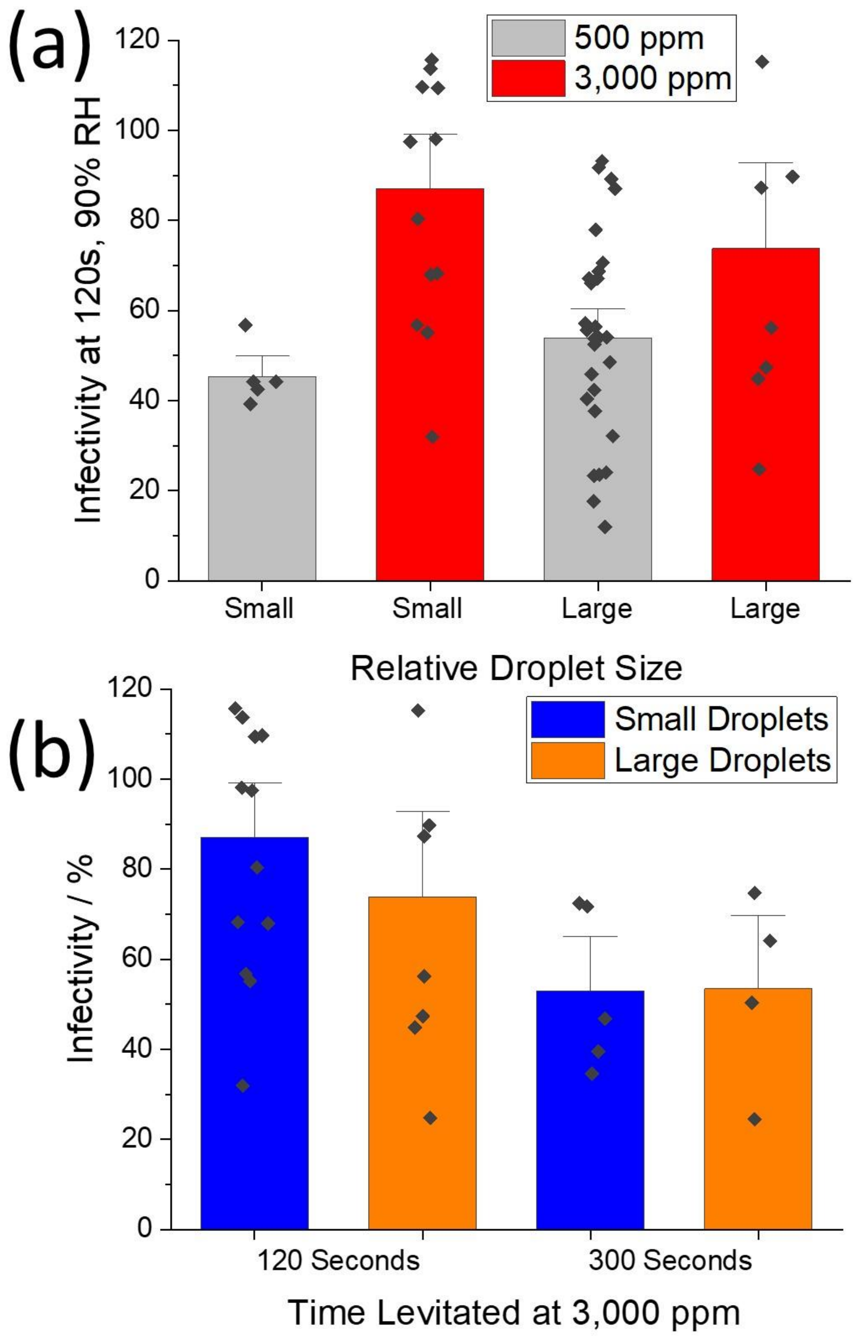

Supplemental Figure 6: Effect of initial droplet size on the interplay between viral decay and elevated [$CO_{2(g)}$]. Based upon the viral tire per droplet, the initial volume of the "Small" droplets (0.6 infectious viral units per droplet) were approximately a quarter of the "Large" droplets (2.8 infectious viral units per droplet). (a) At 120s, increasing the [$CO_{2(g)}$] was found to have a similar effect on both droplet sizes. n = 54 independent measurements. (b) Over time, the effect of the increased [$CO_{2(g)}$] was found to be similar for both particle sizes. n = 31 independent measurements.

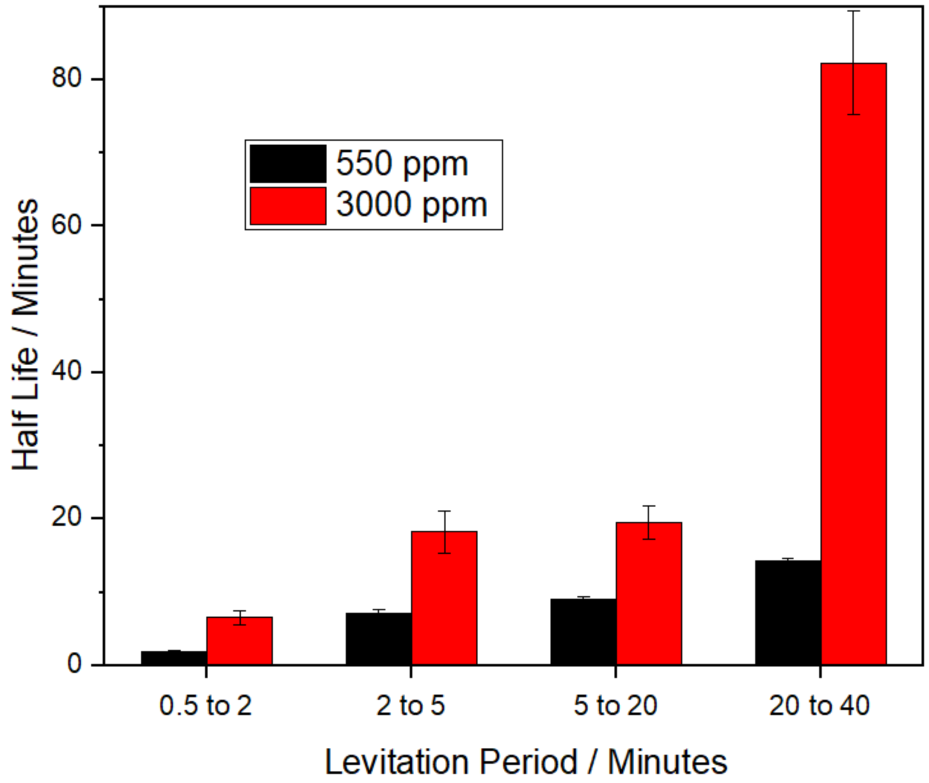

Supplemental Figure 7: Estimated half-life of SARS-CoV-2 as a function of the time range in which the decay rate is measured, and of the [CO_2].

Supplemental References

1 Dawes, C. The effects of flow rate and duration of stimulation on the condentrations of protein and the main electrolytes in human parotid saliva. *Arch Oral Biol* **14**, 277-294 (1969). https://doi.org:10.1016/0003-9969(69)90231-3

2 Lasisi, T. J. & Ugwuadu, P. N. Pregnancy related changes in human salivary secretion and composition in a Nigerian population. *Afr J Med Med Sci* **43**, 347-351 (2014).

3 Haddrell, A., Rovelli, G., Lewis, D., Church, T. & Reid, J. Identifying time-dependent changes in the morphology of an individual aerosol particle from its light scattering pattern. *Aerosol Sci Tech* **53**, 1334-1351 (2019). https://doi.org:http://10.1080/02786826.2019.1661351

4 Oswin, H. P. *et al.* The dynamics of SARS-CoV-2 infectivity with changes in aerosol microenvironment. *Proc Natl Acad Sci U S A* **119**, e2200109119 (2022). https://doi.org:10.1073/pnas.2200109119
