# Supplementary material for: Ambient carbon dioxide concentration correlates with SARS-CoV-2 aerostability and infection risk
Source: Nat Commun. 2024 Apr 25;15:3487. doi: 10.1038/s41467-024-47777-5 (PMC11045827; doi:10.1038/s41467-024-47777-5)
Supplement: Supplementary file 3 — Reporting Summary [file 41467_2024_47777_MOESM3_ESM.pdf]

nature portfolio

NCOMMS-23-35569B
Corresponding author(s): Allen Haddrell

Last updated by author(s): Mar 14, 2024

Reporting Summary

Nature Portfolio wishes to improve the reproducibility of the work that we publish. This form provides structure for consistency and transparency in reporting. For further information on Nature Portfolio policies, see our Editorial Policies and the Editorial Policy Checklist.

Statistics

For all statistical analyses, confirm that the following items are present in the figure legend, table legend, main text, or Methods section.

n/a	Confirmed	
☐	☒	The exact sample size (*n*) for each experimental group/condition, given as a discrete number and unit of measurement
☐	☒	A statement on whether measurements were taken from distinct samples or whether the same sample was measured repeatedly
☐	☒	The statistical test(s) used AND whether they are one- or two-sided<br>*Only common tests should be described solely by name; describe more complex techniques in the Methods section.*
☒	☐	A description of all covariates tested
☐	☒	A description of any assumptions or corrections, such as tests of normality and adjustment for multiple comparisons
☐	☒	A full description of the statistical parameters including central tendency (e.g. means) or other basic estimates (e.g. regression coefficient) AND variation (e.g. standard deviation) or associated estimates of uncertainty (e.g. confidence intervals)
☐	☒	For null hypothesis testing, the test statistic (e.g. *F*, *t*, *r*) with confidence intervals, effect sizes, degrees of freedom and *P* value noted<br>*Give P values as exact values whenever suitable.*
☒	☐	For Bayesian analysis, information on the choice of priors and Markov chain Monte Carlo settings
☒	☐	For hierarchical and complex designs, identification of the appropriate level for tests and full reporting of outcomes
☒	☐	Estimates of effect sizes (e.g. Cohen's *d*, Pearson's *r*), indicating how they were calculated

Our web collection on statistics for biologists contains articles on many of the points above.

Software and code

Policy information about availability of computer code

Data collection	In house LabView, Microsoft Excel
Data analysis	Microsoft Excel, GraphPad Prism v9.4.0

For manuscripts utilizing custom algorithms or software that are central to the research but not yet described in published literature, software must be made available to editors and reviewers. We strongly encourage code deposition in a community repository (e.g. GitHub). See the Nature Portfolio guidelines for submitting code & software for further information.

Data

Policy information about availability of data

All manuscripts must include a data availability statement. This statement should provide the following information, where applicable:

- Accession codes, unique identifiers, or web links for publicly available datasets
- A description of any restrictions on data availability
- For clinical datasets or third party data, please ensure that the statement adheres to our policy

There are no restrictions to accessing the data set. Data are available at the University of Bristol data repository, data.bris, at https://doi.org/10.5523/bris.17xvyth00473q2cxnj3ubg1vm7

Research involving human participants, their data, or biological material

Policy information about studies with human participants or human data. See also policy information about sex, gender (identity/presentation), and sexual orientation and race, ethnicity and racism.

Reporting on sex and gender	n/a
Reporting on race, ethnicity, or other socially relevant groupings	n/a
Population characteristics	n/a
Recruitment	n/a
Ethics oversight	n/a

Note that full information on the approval of the study protocol must also be provided in the manuscript.

Field-specific reporting

Please select the one below that is the best fit for your research. If you are not sure, read the appropriate sections before making your selection.

☐ Life sciences ☐ Behavioural & social sciences ☒ Ecological, evolutionary & environmental sciences

For a reference copy of the document with all sections, see nature.com/documents/nr-reporting-summary-flat.pdf

Ecological, evolutionary & environmental sciences study design

All studies must disclose on these points even when the disclosure is negative.

Study description	The length of time that different variants of SARS-COV-2 remained infectious in the aerosol phase as a function of environmental conditions, including temperature, relative humidity and [CO2]. Small populations of near identical particles were levitated using a novel technology, and the percentage of virus to survive the levitation process (as a function of time, droplet composition, and gas phase composition) was measured. Through systematically changing these parameters, the effect the aerosol pH has on the loss of viral decay was explored. For each time point/condition, biological replicates were made in at least sets of three
Research sample	Proxies for respiratory aerosol containing SARS-CoV-2 were studied; the virus was originally collected from infected individuals, and stocks were grown in cell culture (Vero E6, isolated from the kidney of an African green monkey). These were used as they mimic the real world pathogenic aerosol. In each individual experiment, a single sample would be the population of less than 30 individual droplets that were probed. The total number of infectious viral units in the aerosol are meant to represent/replicate the relative number of infectious viral particles in an exhaled breath, where the percentage drop in infectivity for both will be similar.
Sampling strategy	In each individual measurement, a population of less than 30 individual viral containing particles are probed; the are sampled using a droplet electrodynamic levitation technology (CELEBS). The reason for this population size was the physical limitation of the levitation device used. It is very difficult to both hold and quantify the number of levitated particle when the population is over 30; to improve reproducibility, smaller populations were probed.
Data collection	The percentage viral particles that survived the levitation was quantified through microscopic analysis, where the absolute number of viral particles that survived the levitation process was indicated by the characteristic cytopathic effect. The data was recorded by hand by the experimentalist (Haddrell, Otero-Fernandez and Oswin).
Timing and spatial scale	The data collection period of this study went from October 2022 through to May 2023. Levitations were undertaken 2 to 3 days of the week, and the infectivity assay was done once a week. This periodicity was selected as it allowed for the maximum amount of days spent levitating.
Data exclusions	No data were excluded.
Reproducibility	Measurements were repeated on the same day to account for variability in the droplet generation process and Poisson effects, and on separate days to verify the reproducibility across different cell culture batches, stocks of virus culture. Visual verification of the droplet levitation/deposition process ensured that the attempts to repeat the experiment were successful. On occasion, droplet deposition would be observed to fail during the deposition process; those samples were excluded.
Randomization	In this microbiology based study, the virus samples were grown in bulk and thus assumed to be homogeneous throughout. During the levitation studies, the levitation times were varied to ensure that the changes in decay rate were due to the conditions in the levitation chamber and not the experimental design or changes in the stock concentration throughout the day.<br><br>For example, if the survival at 1, 5, 20 and 40 minutes was probed (in triplicate), they would not be done in order. In this example, the sequence of levitation measurements would be something like: 1, 5, 40, 1, 20, 40, 5, 20, 1, 5, 40, 20.

	The precise number of droplets per sample (where a sample is the population of <30 viral containing droplets) were varied to increase randomness in the data. The number of samples per time point/condition were also varied due to logistical reasons such as the number of 96 well plates (used to quantify the number of infectious viral particles) accessible each day.
Blinding	During the quantification of the remaining infectious viral load, the identity of the conditions of the sample were not known until after the number of surviving viral particles were collected.

Did the study involve field work? ☐ Yes ☒ No

Reporting for specific materials, systems and methods

We require information from authors about some types of materials, experimental systems and methods used in many studies. Here, indicate whether each material, system or method listed is relevant to your study. If you are not sure if a list item applies to your research, read the appropriate section before selecting a response.

Materials & experimental systems

n/a	Involved in the study
☐	☒ Antibodies
☐	☒ Eukaryotic cell lines
☒	☐ Palaeontology and archaeology
☒	☐ Animals and other organisms
☒	☐ Clinical data
☒	☐ Dual use research of concern
☒	☐ Plants

Methods

n/a	Involved in the study
☒	☐ ChIP-seq
☒	☐ Flow cytometry
☒	☐ MRI-based neuroimaging

Antibodies

Antibodies used	- Anti-SARS-CoV Nucleocapsid (N) Protein (RABBIT) Antibody (200-401-A50, Rockland, Lot #46527)<br>- Alexa Fluor-conjugated secondary antibody (ThermoFisher)
Validation	- Anti-SARS-CoV Nucleocapsi: This protein A purified antibody has been tested for use in ELISA, western blot, Immunohistochemistry, Immunofluorescence, and lateral flow. https://www.rockland.com/globalassets/CofA/200-401-A50_46527<br>- Alexa Fluor-conjugated secondary antibody (ThermoFisher).

Eukaryotic cell lines

Policy information about cell lines and Sex and Gender in Research

Cell line source(s)	Vero E6 cells modified to stably express TMPRSS2 (Vero E6/TMPRSS2 cell, obtained from NIBSC, UK) and Vero E6 cells modified to stably express human ACE2 and TMPRSS2 (Vero E6/ACE2/TMPRSS2 (VAT) cells (obtained from the Centre of Virus Research, University of Glasgow).
Authentication	Cells lines were not authenticated by NIBSC, UK.
Mycoplasma contamination	Cell lines were not routinely tested for mycoplasma contamination. Rather, fresh lines were grown approximately every two months from frozen stocks that had been mycoplasma tested.
Commonly misidentified lines<br>(See ICLAC register)	No commonly misidentified cell lines were used in this study.
